# Machine learning to predict virological failure among HIV patients on antiretroviral therapy in the University of Gondar Comprehensive and Specialized Hospital, in Amhara Region, Ethiopia, 2022

**DOI:** 10.1186/s12911-023-02167-7

**Published:** 2023-04-21

**Authors:** Daniel Niguse Mamo, Tesfahun Melese Yilma, Makda Fekadie Tewelgne, Yakub Sebastian, Tilahun Bizuayehu, Mequannent Sharew Melaku, Agmasie Damtew Walle

**Affiliations:** 1 Department of Health Informatics, College of Medicine and Health Sciences, School of Public Health, Arbaminch University, Arbaminch, Ethiopia; 2 Department of Health Informatics, Institute of Public Health, University of Gondar, Gondar, Ethiopia; 3 College of Engineering, IT, and Environment, Charles Darwin University, Casuarina, Australia; 4 Department of Internal Medicine, School of Medicine, University of Gondar, Gondar, Ethiopia; 5 Department of Health Informatics, college of health science, Mettu University, Mettu, Ethiopia

**Keywords:** *HIV/AIDS*, *Virological failure*, *Machine learning*, *Antiretroviral treatment*, *Ethiopia*

## Abstract

**Background:**

Treatment with effective antiretroviral therapy (ART) reduces viral load as well as HIV-related morbidity and mortality in HIV-positive patients. Despite the expanded availability of antiretroviral therapy around the world, virological failure remains a serious problem for HIV-positive patients. Thus, Machine learning predictive algorithms have the potential to improve the quality of care and predict the needs of HIV patients by analyzing huge amounts of data, and enhancing prediction capabilities. This study used different machine learning classification algorithms to predict the features that cause virological failure in HIV-positive patients.

**Method:**

An institution-based secondary data was used to conduct patients who were on antiretroviral therapy at the University of Gondar Comprehensive and Specialized Hospital from January 2020 to May 2022. Patients’ data were extracted from the electronic database using a structured checklist and imported into Python version three software for data pre-processing and analysis. Then, seven supervised classification machine-learning algorithms for model development were trained. The performances of the predictive models were evaluated using accuracy, sensitivity, specificity, precision, f1-score, and AUC. Association rule mining was used to generate the best rule for the association between independent features and the target feature.

**Result:**

Out of 5264 study participants, 1893 (35.06%) males and 3371 (64.04%) females were included. The random forest classifier (sensitivity = 1.00, precision = 0.987, f1-score = 0.993, AUC = 0.9989) outperformed in predicting virological failure among all selected classifiers. Random forest feature importance and association rules identified the top eight predictors (Male, younger age, longer duration on ART, not taking CPT, not taking TPT, secondary educational status, TDF-3TC-EFV, and low CD4 counts) of virological failure based on the importance ranking, and the CD-4 count was recognized as the most important predictor feature.

**Conclusion:**

The random forest classifier outperformed in predicting and identifying the relevant predictors of virological failure. The results of this study could be very helpful to health professionals in determining the optimal virological outcome.

## Background

According to the Global HIV/AIDS Statistics, around 38 million individuals are living with HIV, 28.7 million people were accessing antiretroviral therapy, 1.5 million persons are newly infected with HIV, and 650,000 people died from AIDS-related illnesses around the world in 2021 [1]. Approximately 67% of HIV-positive people live in Africa [2]. In Eastern and Southern Africa, 20.6 million people are living with HIV, and 16.2 million individuals receiving antiretroviral therapy [1]. UNAIDS hoped that by the end of 2020, 90% of people living with HIV knew their HIV status, 90% of those who knew their HIV-positive status were on treatment, and 90% of those on treatment had suppressed viral load [1]. Since this target was not achieved, UNAIDS modified it to 95-95-95 targets to end the HIV/AIDS pandemic by 2030 [3]. Nonetheless, it remains a serious global public health issue; 40 million people have died from AIDS-related illnesses since the epidemic began [4].

Furthermore, Ethiopia is one of the most affected countries in Sub-Saharan Africa by the HIV epidemic, with an estimated 617,921 people living with HIV, 78% of adults and children receiving ART. More than 60% of new infections occur in the Amhara, Oromia, SNNP, and Tigray regions, with the Amhara region having the highest number of HIV patients at around 191,480. The prevalence of HIV among adults in Ethiopia is 0.9% [5–7]. According to the Ethiopian National HIV Care Guideline, virological failure is defined as a viral load greater than 1000 copies/ml based on two consecutive viral load tests in three months, with improved adherence support following the first viral load test [8]. This guideline is approved by WHO that viral load monitoring is the best standard for determining the efficacy of treatment [9]. Virological failure is a common problem encountered by HIV patients after the initiation of treatment [10]. WHO guidelines recommend that virological failure should not exceed 10% after the initiation of treatment [10, 11]. However, studies show that viral failure is much higher than expected, and viral replication remains a major challenge for HIV-infected patients [12–14]. Patients who experienced virologic failure were at increased risk of clinical progression to AIDS and death compared with patients who had a complete virological response [15].

In Sub-Saharan African countries, HIV viral load testing has been used and scaled up to monitor patients on antiretroviral therapy (ART). However, more than 24% of patients receiving first-line ART experience virologic failure within 1 year of starting therapy [16, 17]. Failure to achieve and maintain viral suppression can lead to resistance and an increased risk of viral infection [17, 18]. Therefore, with delayed early identification of virological failure, patients with HIV are more likely to have illness progression and death [2, 19, 20]. As a result, effective antiretroviral therapy reduces the incidence and mortality in HIV-positive patients [4]. Furthermore, HIV viral load suppression is the most important indicator of successful antiretroviral therapy, progression, and death, as well as the most effective monitoring strategy for confirming a diagnosis and proving ARV treatment failure [21]. Despite the expanded availability of antiretroviral treatment (ART) around the world, virological failure remains a significant challenge for HIV patients [22, 23]. Delays in commencing ART can have serious consequences, particularly for people with tuberculosis or extensive immunosuppression who are at high risk of mortality [21, 24]. A study indicates that viral suppression was much lower than expected at the first test, and viral replication remains a major issue for HIV patients [25]. According to recent research conducted in Sub-Saharan Africa, the proportion of individuals who experienced virological failure ranged from 11 to 47% [26–29]. Furthermore, in Ethiopia, studies reported that virologic failure rates ranged from 5.4 to 19% [30–32]. Several studies have found that socio-demographic predictors (age, gender, marital status, religion), clinical-related predictors (low BMI, TB co-infection), and treatment-related predictors (excessive dose frequency, poor medication adherence) can all influence virological failure in HIV patients [30, 33–36]. However, the predictors of virological failure varied between studies.

Understanding the causes that lead to virological failure in HIV patients on antiretroviral therapy (ART) is essential for HIV/AIDS prevention and control programs Also minimize these adverse outcomes, clinical prediction models that use patient-level evidence can be used in medical decision-making [37]. Meanwhile, traditional statistical modeling was created for data with a few dozen input variables and sample sizes that would be considered small to medium. The complexity of data may make traditional statistical inference less manageable. As a result, machine learning was used more effectively [38]. Machine learning methods can identify and discover patterns in complex datasets and predict future HIV treatment outcomes with excellent predictive performance [39–41]. Machine learning predictive algorithms have the potential to improve the quality of care and predict the needs of HIV patients by analyzing huge amounts of data, and enhancing prediction capabilities [42, 43]. Furthermore, machine learning is used to identify HIV patients who are at high risk of failing to adhere and being a virological failure [44] and able to learn from domain experts quickly and accurately, then apply that knowledge to identify HIV risk behaviors in a huge dataset [45]. Ethiopia has a severe HIV pandemic with increasing virological failure [46]. Despite these facts, researchers using data from this country have only used statistical methods to identify factors of viral failure [46–48]. Although previous studies have been conducted to investigate the determinants of virological failure in different regions of Ethiopia, most of them have used classical statistical models to identify important predictors of virological failure. Therefore, advanced predictive modeling is required to improve the targeting of interventions through differentiated care models, to be more cost-effective, to improve patient outcomes, and to initiate treatment based on achieving maximum patient survival and clinical benefit. Therefore, this study took advantage of the unique opportunity provided by access to the secondary dataset in the ART clinic of the University of Gondar comprehensive and specialized hospital. Then, we compare the relative performances of various machine learning algorithms for predicting virological failure using ART datasets. Identifying the risk factors for virological failure in HIV-positive patients also helps to prevent the virus from spreading and reduces the possibility of treatment failure.

## Method

### Study design

This study used a quantitative research method with a machine learning approach and an institution-based cross-sectional study was conducted at the University of Gondar Comprehensive and Specialized Hospital.

### Study area and period

This study was conducted at the University of Gondar Comprehensive and Specialized Hospital using secondary data from January 2020 to May 2022. The University of Gondar Comprehensive and Specialized Hospital is located in the Amhara National Regional State, Gondar City Administration. It is located 748 km northwest of Ethiopia’s capital, Addis Ababa. The hospital, which is Ethiopia’s oldest academic institution, is a teaching hospital that provides teaching activities to medical and health science students. The hospital provides medical education, training, medical services, and many other services to more than 7 million people in Gondar province and neighboring areas. One of the services provided is the ART service, and through its service, both children and adults can get a free diagnosis, treatment, and monitoring. The hospital started a free ART service in March 2005. At the University of Gondar hospital, the ART clinic reported that 15,933 patients were enrolled for ART. Of those, 5523 patients are actively taking the treatment.

### Source population and study population

All adult (≥ 18 years) HIV-positive patients on antiretroviral therapy (ART) who attend ART clinics at the University of Gondar Comprehensive and Specialized Hospital were the source population, while all adult HIV-positive patients who have been on antiretroviral therapy (ART) for at least 6 months at the University of Gondar Comprehensive and Specialized Hospital were the study population.

### Inclusion and exclusion criteria

All adult HIV-positive patients who have been on antiretroviral therapy (ART) for at least 6 months, have had viral load tests, and have records in the ART computerized database at the University of Gondar Comprehensive and Specialized Hospital were included. However, the records that had significant missing feature values (like CD4 counts, Adherence, and WHO Stage) were excluded.

### Sample size and sampling procedure

Out of all the ART patients who received treatment at the time this study was undertaken, 5264 adult HIV-positive patients from the University of Gondar Comprehensive Specialized Hospital’s ART clinic were included for the model building and prediction of virological failure.

### Study variables

#### Dependent and independent variables

In this study, the dependent variable was a virological failure while the independent variables were sociodemographic factors (age, sex, marital status, educational status, residence), clinical-related factors (BMI, WHO stages, TB screened, TB screened result, functional status), hematologic and immunological factors (CD4 count) and treatment-related factors (treatment adherence, duration on ART, ART regimen, TPT started, TPT discontinued, CPT use, viral load test duration).

#### Operational definition

**Virological failure** is defined as a viral load greater than 1000 copies/ml based on two consecutive viral load tests within three months, with better adherence support after the first viral load test [21]. Using the definition of HIV viral load, this study classified the outcome variable into suppressed (not virological failure) and unsuppressed (virological failure). If the patient’s viral load was less than 1000 copies/ml, the outcome variable was classified as suppressed (not virological failure), and if the viral load was larger than 1000 copies/ml, the outcome variable was classified as unsuppressed (virological failure).

**Treatment adherence** is defined as the percentage of a person’s medication-taking behavior that conforms to agreed-upon recommendations from a healthcare practitioner. Good adherence is defined as more than 95%, fair adherence is defined as between 85% and 94%, and poor adherence is defined as less than 85% adherence [21].

#### Data collection tools and procedures

The main source of data for this study was the ART clinic at the University of Gondar Comprehensive and Specialized Hospital, which had existing record data on an electronic database that contained information on HIV-positive patients. It is identified by unique ART numbers and MRN. From all ART patients, a total of 5,264 adult HIV-positive patients’ data were extracted from the electronic database using a structured checklist that was prepared in English. It was adapted from the Ethiopian Federal Ministry of Health ART clinic intake and follow-up form [49]. Multiple tables with a variety of feature integrations were present in the electronic database. The features were chosen after discussions with subject-matter experts and categorized as sociodemographic features, hematologic and immunological features, clinical-related features, and treatment-related features. Features with identifiable information such as patient names and phone numbers were removed during data extractions. After all data collection procedures were completed, twenty features and one target feature were identified for this study.

#### Data quality assurance

Data quality assurance is an important step for determining the quality of data. During the initial stage of data collection from the electronic database, the principal investigator provided one day of training to the ART data clerk and observed each step of deidentified record extraction for the feature range value consistency and correctness of the collective data. The principal investigator repeatedly checked the data completeness and randomly cross-checked the patient charts to ensure data similarity and consistency.

#### Data management and analysis

Patients’ data were extracted from the electronic database in Microsoft Excel format and then converted to comma-separated values (CSV) to make the dataset easier to pre-process in Python version 3. Machine learning needs a high-quality dataset for prediction. Due to this, handling the missing data during the pre-processing of the dataset is a crucial phase. An imputation technique was applied to handle the missing values in the dataset. As a result, this study handled the missing values using simple imputation techniques. The Simple Imputer class of the scikit-learn module was used for imputing the missing values in the dataset [50]. Data pre-processing also includes encoding data, which is a crucial and essential step. It used one-hot and label encoding to encode categorical variables. Values with two or more category values are considered categorical if they are discrete and not continuous. In this study, categorical variables were encoded using one hot encoding approach. The categorical values are replaced by a number between 0 and 1 in one hot encoding [51].

#### Data Analysis

In this study, descriptive statistics were used to describe the socio-demographic characteristics using frequency and percentage. Some of the data analysis parts were data pre-processing, feature selection, data split, imbalanced data handling, building the model, and testing the performance of the model. In this study, Python version 3 software was used. It is commonly used in data analysis and interactive computing. It has also been a popular choice for data analysis tasks in recent years and It has libraries for data loading, visualization, statistics, natural language processing, and image processing [52, 53].

#### Feature selection method

Feature selection is one of the main processes of data dimensionality reduction in machine learning. It is the method of extracting the necessary beneficial factors from a dataset to improve the accuracy of machine learning predictions [54]. A dataset with redundant, irrelevant, and inconsistent features may be reducing the model’s performance. It is essential to use feature selection methods to improve the accuracy of model performance and reduce the training time and overfitting [55].

In this study, different feature selection methods such as recursive feature elimination (RFE), random forest feature importance, and the Boruta feature selection method were used to select the relevant predictive feature. RFE is a feature selection technique that starts with all the features in the training dataset and removes features until the target number of the feature remains. Furthermore, it is a relatively efficient technique for decreasing model complexity and speeding up the processing of machine-learning algorithms [56].

#### Data split

A typical strategy in machine learning is to split the data into training and testing sets. K-fold cross-validation is commonly used for improved model selection in classification prediction (50 ). This study used the stratified tenfold cross-validation method, which partitioned the entire dataset into ten folds. Nine folds were used to train the model and then the remaining one fold was used to test the model. The procedure is repeated ten times with the testing fold [57, 58]. In addition, cross-validation was a useful method for this study because the available trained dataset was small and the total performance of the model is the average of all 10 folds [59].

#### Imbalance data handling

Many real-world applications, including medical diagnosis, pattern recognition, and fraud detection, rely on imbalanced data learning. The problem occurs when there is a big gap in numbers between the majority and minority classes, which is particularly common in classes having binary values, and frequently occurs in supervised machine learning [60]. The outcome variable for this study was a binary classification of suppressed (not virological failure) and unsuppressed (virological failure). The majority class was suppressed (not virological failure) while the minority class was unsuppressed (virological failure).

Predictive accuracy is commonly used to evaluate the performance of machine learning algorithms, but the imbalance of data affects accuracy and machine learning prediction, making it difficult to determine the cause of virological failure. In this study, the Synthetic Minority Oversampling Technique (SMOTE) [61]. Adaptive Synthetic (ADASYN), and random under-sampling were used to balance the majority and minority classes. SMOTE is an oversampling approach for imbalanced datasets that is used as a preliminary step in learning algorithms and is an effective way of handling class imbalance. It creates new samples by randomly interpolating linearly between a few samples and their neighbors [62, 63]. Random under-sampling is a method of balancing the imbalance of classes in which the majority class is under-sampled by randomly discarding samples from the population of the majority class until the minority class reaches a certain percentage of the majority class [61].

ADASYN is the adaptive generation of minority data samples per their distributions. More synthetic data is generated for minority class samples that are harder to learn compared to those minority samples that are easier to learn and can minimize the bias introduced by the imbalanced data distribution [64]. The figure below shows that to balance the dataset, under-sampling and over-sampling methods [65].

#### Method of building a predictive model

Predictive modeling is defined as “the process of developing a mathematical tool or model that generates an accurate prediction” [66]. Classification algorithms are supervised learning techniques that divide a set of data into specific categories. In this study, seven supervised classification algorithms were applied. Some of the classification machine learning algorithms were support vector machine, random forest, decision tree, logistic regression, gradient boosting, K-nearest neighbours, and XGBoost. During the literature review, it has been observed that these algorithms are better suited for classification problems in the healthcare domains [43, 67–69]. The algorithms were chosen based on their accuracy, training duration, ability to handle missing data, and ease of interpretation and learning.

#### Performance evaluation for predictive model

After model training, the model’s performances are evaluated and compared to each other. The performance of the prediction models was evaluated based on the confusion matrix. This study used precision, sensitivity, specificity, F1-score, and the area under the receiver-operating characteristic (AUC-ROC) to assess the model performance. AUC-ROC is a popular and powerful performance metric for assessing the performance of binary classifiers and is used to evaluate the model’s predictive classification capacity. A range of 0.5 indicated no ability to predict, while 1.0 indicated exceptional or perfect predictive ability [44, 70]. The confusion matrix, which is used to represent the output of a model as a binary class, is a standard performance metric tool used in machine learning classification tasks. The confusion matrix was crucial for this study to determine the sensitivity, precision, f1-score, and performance metrics of accuracy. The matrix is made up of predictions that have been summarized into a total number of correct and incorrect predictions [70, 71] (Table 1).

Equations used to calculate Recall (sensitivity), (specificity), precision, and accuracy are given below.

1 $$\mathrm{Recall}\;(\mathrm{Sensitivity},\;\mathrm{true}\;\mathrm{positive}\;\mathrm{rate})\;=\frac{\mathrm{TP}}{\mathrm{TP}+\mathrm{FN}}$$

2 $$\mathrm{Specificity}\;(\mathrm{true}\;\mathrm{negative}\;\mathrm{rate})\;\;=\frac{\mathrm{TN}}{\mathrm{TN}+\mathrm{FP}}$$

3 $$\mathrm{Precision}\;(\mathrm{positive}\;\mathrm{predictive}\;\mathrm{value})\;\;=\frac{\mathrm{TP}}{\mathrm{TP}+\mathrm{FN}}$$

4 $$\mathrm{Accuracy}=\frac{\mathrm{TP}+\mathrm{FN}}{\mathrm{TN}+\mathrm{TP}+\mathrm{FP}+\mathrm{FN}}$$

5 $$\mathrm F1\;=2\ast\frac{\mathrm{Recall}\ast\mathrm{Precision}}{\mathrm{Recall}+\mathrm{Precision}}$$

**Table 1:** Confusion matrix show to summarizes the total number of correct and incorrect predictions and actual positive and negative

	Predictive negative		Predictive positive
Actual negative	True Negative (**TN**)	False Positive (**FP**)	
Actual positive	False Negative (**FN**)	True Positive (**TP**)	

From the above confusion matrix, the term was described as:

**True positives (TP)**: the actual true positives that were correctly predicted as true positives

**True negatives (TN): **the actual true negatives that were correctly predicted as true negatives

**False negatives (FN)**: the actual positives that were incorrectly predicted as false negatives

**False positives (FP)**: The actual negatives that were incorrectly predicted as positives

**Fig. 1:**
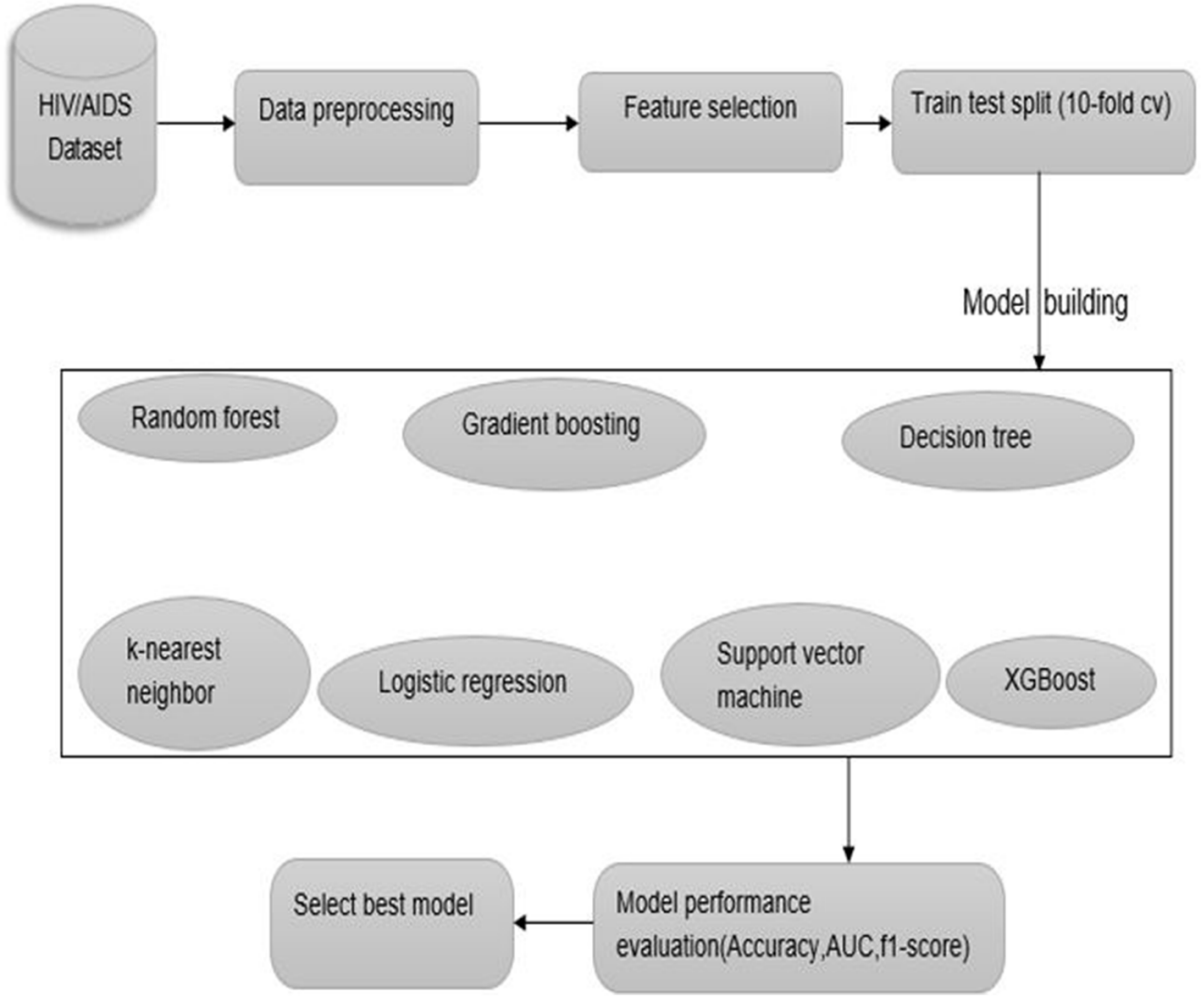
Workflow of machine learning for virological failure prediction

In summary, the machine learning pipeline in this study is depicted in Fig. 1.

#### Association rule

The association rule mining technique, which examines the concurrent correlations between several variables in a grouping, was first created by Agarwal

and Srikanth [72]. Therefore, this study used an additional method that supports the classification of machine learning algorithms for predicting virological failure using R software. Using the apriori algorithm [73] to discover the association between the selected features and the target feature. To identify all potential association rules, the minimal support degree was set at 0.00095 and the minimum confidence threshold at 90% because a rule is considered reliable if its confidence level is more than 80% [74].

To produce association rules, this study primarily focused on features that are implied by the target features (Antecedent = > Consequent), which is a way to classify all the variables that contribute to virological failure. These rules are often referred to as classification association rules [75]. The reason for using this method was to know the predictors that each category contributed to the virological failure.

Support, Confidence, and Lift can be represented as follows for a certain rule: Here, the sets of features represented by X and Y are mutually exclusive.

1 $$\mathrm{Rule}\;\mathrm X=>\mathrm Y$$

2 $$\mathrm{Support}\;=\frac{\mathrm{frequency}(\mathrm X,\mathrm Y)}{\mathrm N}$$

3 $$\mathrm{Confidence}\;=\frac{\mathrm{frequency}(\mathrm X,\mathrm Y)}{\mathrm{frequency}(\mathrm X)}$$

4 $$\mathrm{Lift}\;=\frac{\mathrm{frequency}(\mathrm X,\mathrm Y)}{\mathrm{frequency}(\mathrm X)\mathrm{frequency}(\mathrm Y)}$$

## Results

### Sociodemographic characteristics of participants

From the total number of HIV patients who had ever started antiretroviral therapy, this study includes 5,264 patients who were on antiretroviral therapy. 64% (3371) were females, and 39.31% (2,069) were between the ages of 38 and 47. More than half of HIV patients 57.26% (3014) were married and 77.79% (4095) lived in Gondar city. Almost half of the patients had secondary educational status, and 91.76% (4830) of all patients were orthodox (Table 2).

**Table 2:** Sociodemographic characteristics of the study participants in the University of Gondar Comprehensive and Specialized Hospital, July 2022 (*n* = 5264)

Features	Category	Frequency	Percent (%)
Sex	Female	3371	64.04
	Male	1893	35.96
Age in years			
	18–27	434	8.24
	28–37	1160	22.04
	38–47	2069	39.31
	48–57	1167	22.17
	58 and above	434	8.24
Residence			
	In Gondar	4095	77.79
	Outside Gondar	1169	22.21
Marital Status			
	Never Married	686	13.03
	Married	3014	57.26
	Widowed	535	10.16
	Divorced	1029	19.55
Educational Level			
	No education	1134	21.54
	Primary	1275	24.22
	Secondary	2275	43.22
	Tertiary	580	11.02
Religion			
	Orthodox	4830	91.76
	Catholic	12	0.23
	Protestant	37	0.70
	Muslim	376	7.14
	Other	9	0.17

### Data Pre-processing Results

**Fig. 2:**
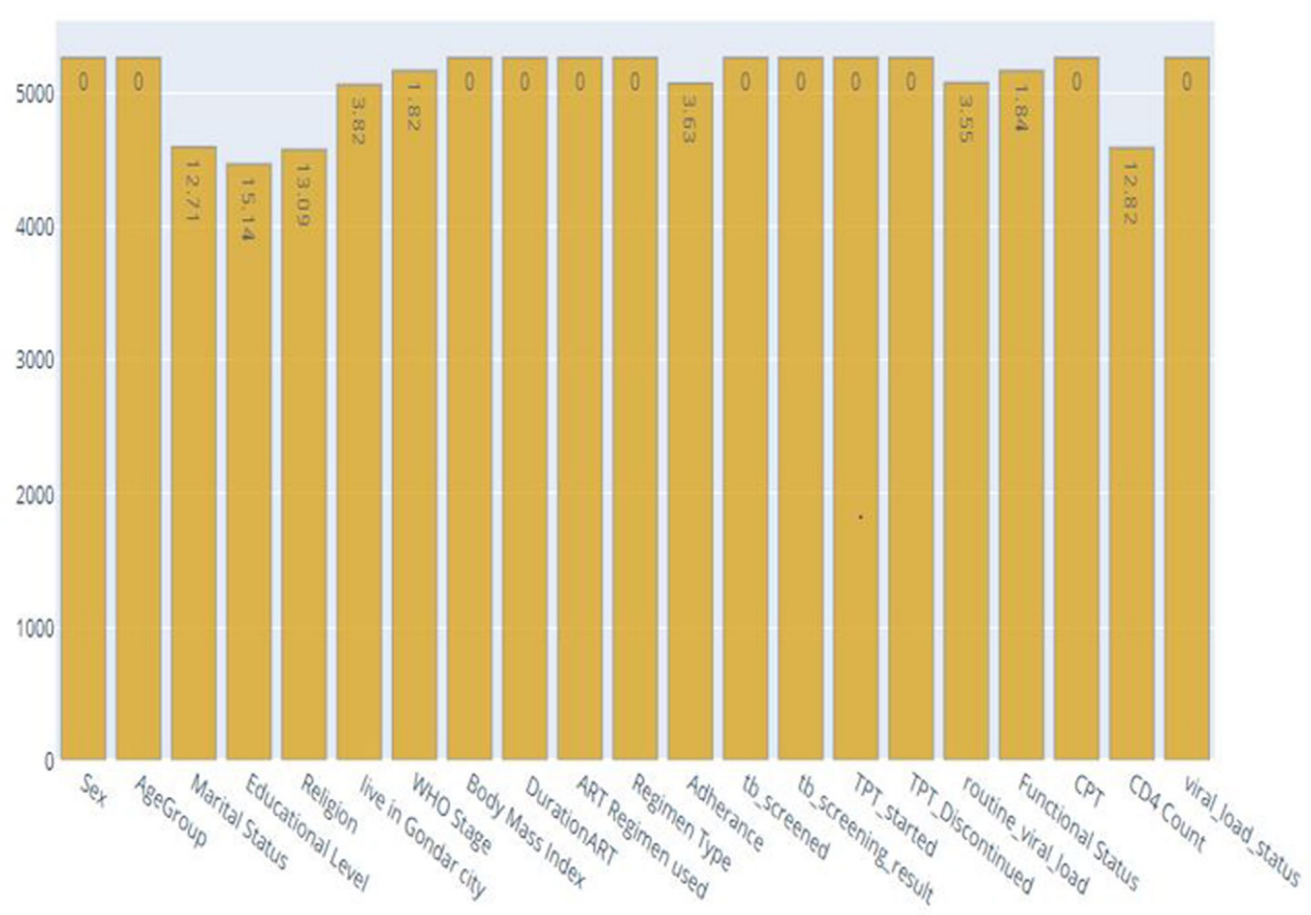
Percentage of missing values in the ART dataset

In this study, the main pre-processing steps involved dealing with missing or null values, encoding categorical labels, and balancing the dataset. Due to the incompleteness of the initial raw data, missing values were handled by the imputation technique. Figure 2 shows the percentage of missing values for each feature. The features with the higher percentage of missing data were educational level (15.14%), religion (13.09%), CD4 count (12.82%), and marital status (12.71%). The missing values were then imputed using the simple imputation technique. Input and output features must be numerical to be used with machines. This implies that to fit our data to the model, we used one hot encoder to encode categorical variables that are present in the dataset. From this, the dataset had twenty features, with one target feature. One-hot encoding is a useful encoding technique for classification tasks. Each categorical value was transformed into a new column with one-hot encoding, and the label values were created as new columns (1 or 0) [51].

### Imbalance data handling

To address the data imbalance issue and increase the effectiveness of the machine learning algorithms, imbalance data handling was an important method for this study. From the outcome feature, 5123 (97.32%) of the observations were classified as suppressed and 141 (2.68%) were unsuppressed. This work used under-resampling, SMOTE, and ADNYSN as balanced sampling methods to apply the classification algorithm to a balanced dataset. Each balanced sampling method used a different sampling technique to balance the unequal dataset by either maximizing the minority class or decreasing the majority class. This study compared the balancing technique with the accuracy and AUC using selected supervised classification machine learning algorithms. In the unbalanced dataset, logistic regression performed with a higher AUC of 0.915 as compared to another classifier, and random forest achieved a higher accuracy of 97.42%. In the ADASYN, the XGBoost classifier had a higher accuracy of 96.24% and an AUC of 0.994. Additionally, support vector machines performed better in under-sampling techniques, with training accuracy and AUC values of 84.60% and 0.94, respectively (Table 3).

**Table 3:** compares imbalanced data handling techniques using accuracy and Area under the curve (AUC)

Algorithms	Comparison method	Unbalanced	SMOTE	Under-Sampling	ADASYN
**Logistic Regression**	Accuracy (%)	97.23	95.11	82.94	94.40
	AUC	**0.915**	0.987	0.913	0.986
** K Nearest Neighbours**	Accuracy (%)	97.34	95.54	81.38	93.20
	AUC	0.705	0.986	0.881	0.973
**Decision Tree**	Accuracy (%)	95.82	98.08	81.83	93.34
	AUC	0.619	0.982	0.817	0.935
**Random Forest**	Accuracy (%)	**97.42**	**98.80**	83.34	95.10
	AUC	0.892	**0.999**	0.917	0.991
**Gradient Boosting**	Accuracy (%)	97.17	95.22	84.52	93.06
	AUC	0.903	0.988	0.912	0.982
**XGBoost**	Accuracy (%)	96.98	95.25	82.58	**96.24**
	AUC	0.870	0.997	0.901	**0.994**
**Support Vector Machine**	Accuracy (%)	97.32	96.99	**84.60**	95.19
	AUC	0.867	0.995	**0.940**	0.991

***AUC ***Area Under Curve, ***SMOTE ***Synthetic Minority Over-sampling Technique, ***ADASYN ***Adaptive Synthetic. **Underline** and **bold** numbers were the highest score of the classifier

**Fig. 3:**
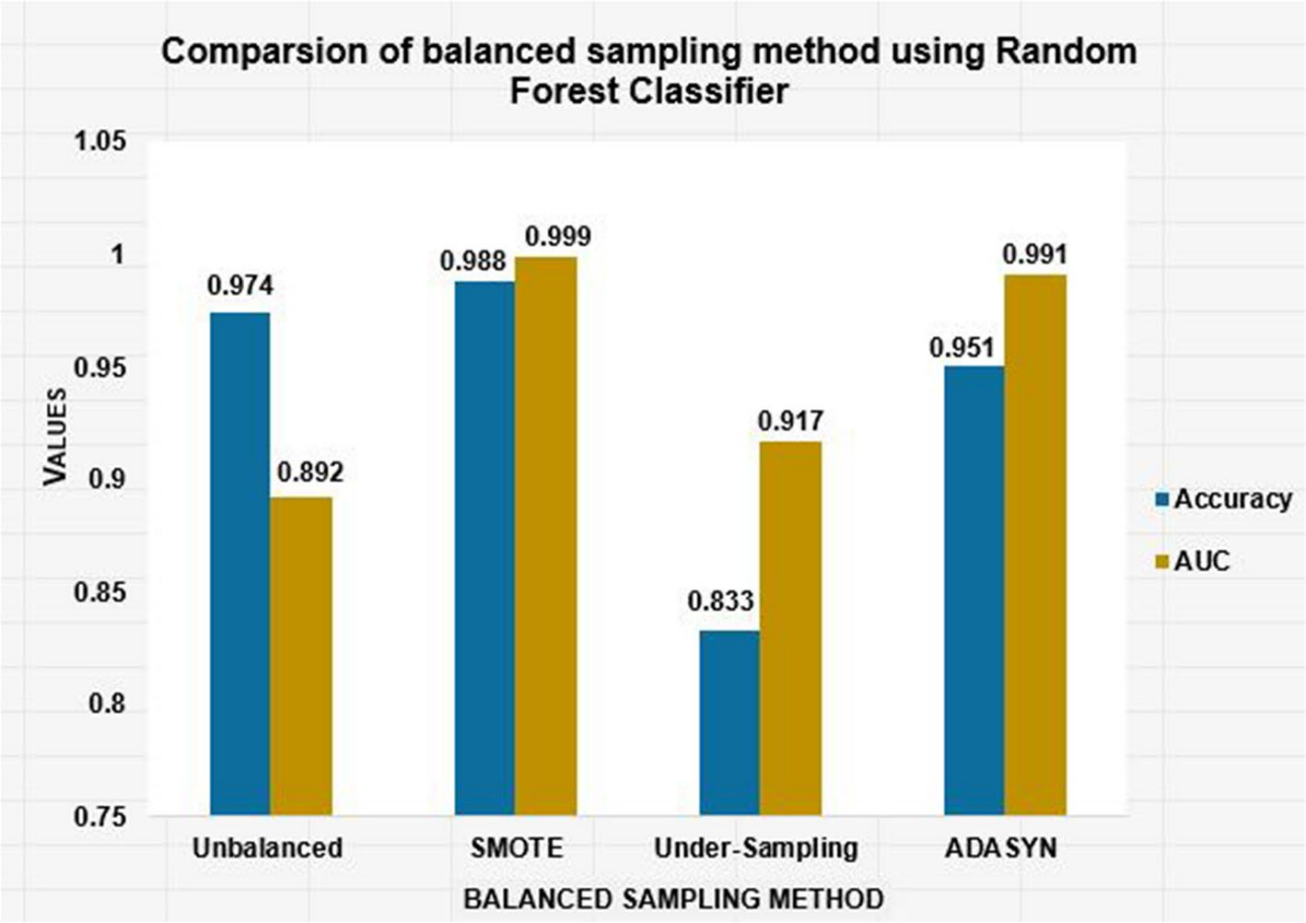
Comparison of balanced sampling method using random forest classifier

In the comparison of different balanced sampling methods using a random forest classifier, the SMOTE sampling balanced method performed better than other sampling methods with an accuracy of 98.80% and an area under the curve (AUC) of 0.999. ADASYN was the second most important balanced resampling method, next to the SMOTE balanced method, with accuracy and an AUC of 95.51% and 0.991, respectively. However, of the balancing imbalanced data handling methods, the under-sampling balanced method was the least important for this study, with an AUC of 0.917 and an accuracy of 83.3% (Fig. 3).

**Fig. 4:**
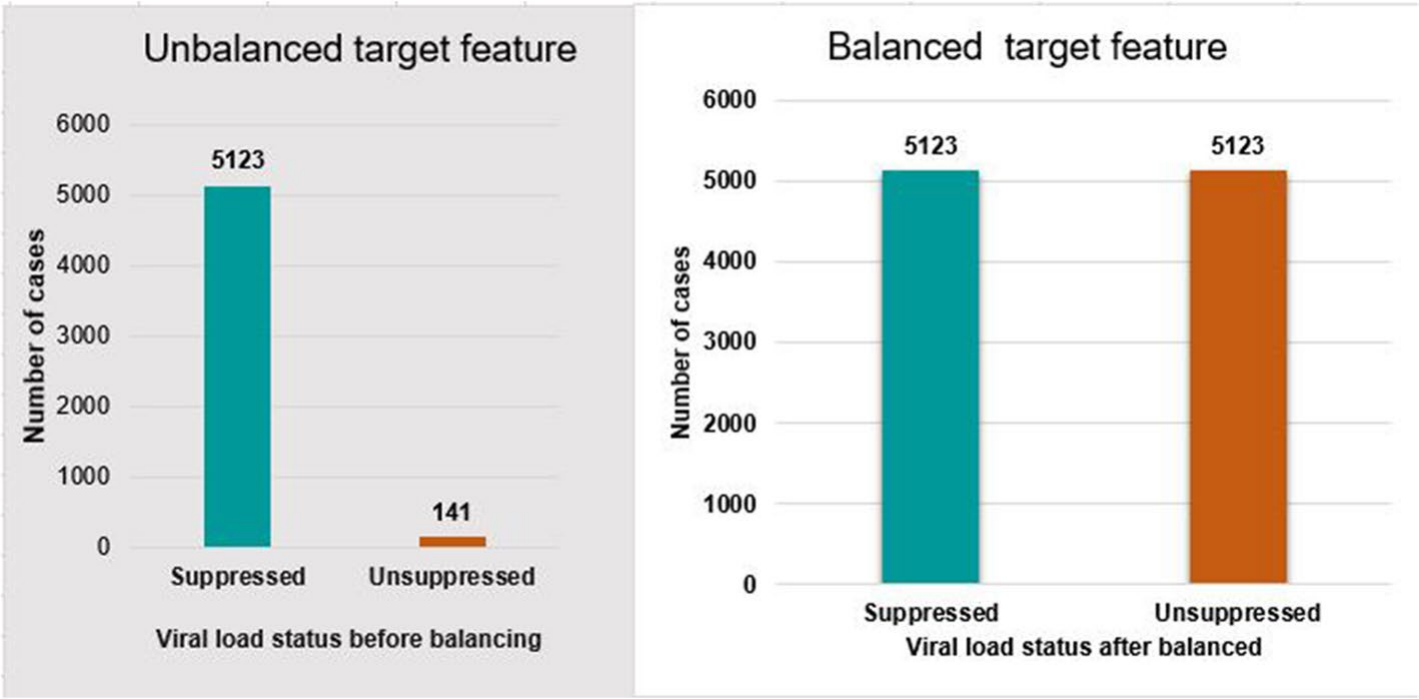
Before unbalanced and after balancing the target feature

Generally, this study had a high issue of imbalanced data. A balanced sampling method was important to overcome such a problem. Unbalanced data makes machine learning challenging and hinders the performance of the classifying algorithm because values from the minority class or rarely occurring classes are mistakenly classified as instances of the majority class. After all, the classifier is overloaded with the majority class and ignores the minority class of virological failure or unsuppressed. The total number of records increased after SMOTE was applied to the unbalanced dataset (Fig. 4). This study compared the classifier and the balanced sampling method with other classifiers using training accuracy and AUC. However, classification algorithms with high accuracy do not always interpret better performance on target datasets because imbalanced data affects the performance of accuracy. For this reason, we mainly used AUC to compare the classifier and balanced sampling method.

### Model building and model evaluation

This study conducted several experiments for model building in predicting the occurrence of unsuppressed or viral failure in patients on ART. These experiments were primarily focused on two parts: the first part trained the various classification algorithms with an unbalanced dataset using twenty features, and the second part used a balanced sampling method to identify the best model with twenty features.

**Fig. 5:**
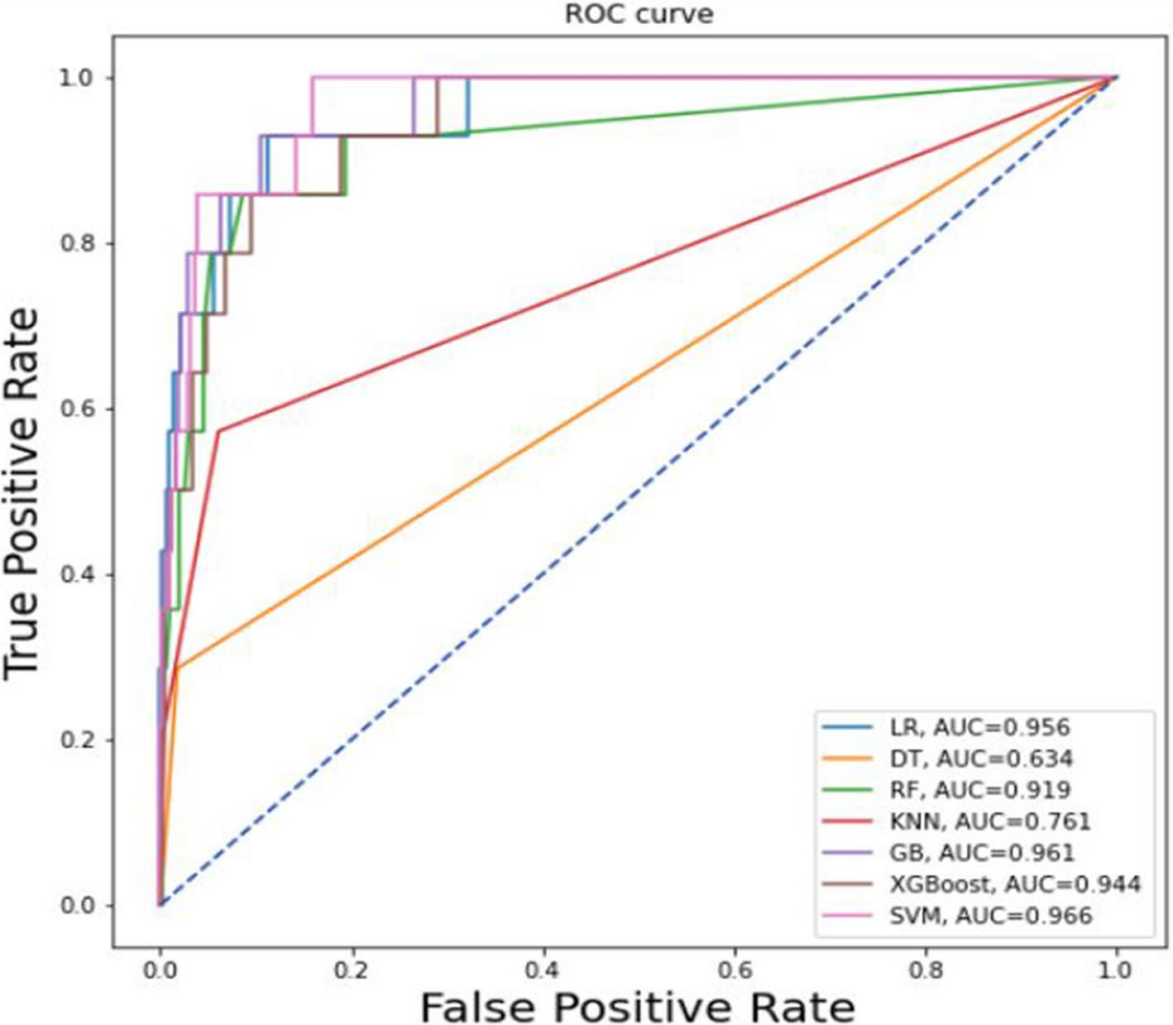
ROC curve shows an unbalanced dataset

The ROC curve in Fig. 5 shows that the imbalanced dataset results from stratified 10-fold cross-validation with default hyperparameter tuning performed less than the balanced dataset from all classifiers. The Support Vector Machine outperformed all other classifiers, with an AUC of 0.966, followed by a gradient boosting classifier with an AUC of 0.961, logistic regression with an AUC of 0.956, XGBoost with an AUC of 0.944, random forest with an AUC of 0.919, and KNN with an AUC of 0.761. Of all the classifiers, the decision tree classifier achieved a lower performance with an AUC of 0.634 (Fig. 5**)**.

**Fig. 6:**
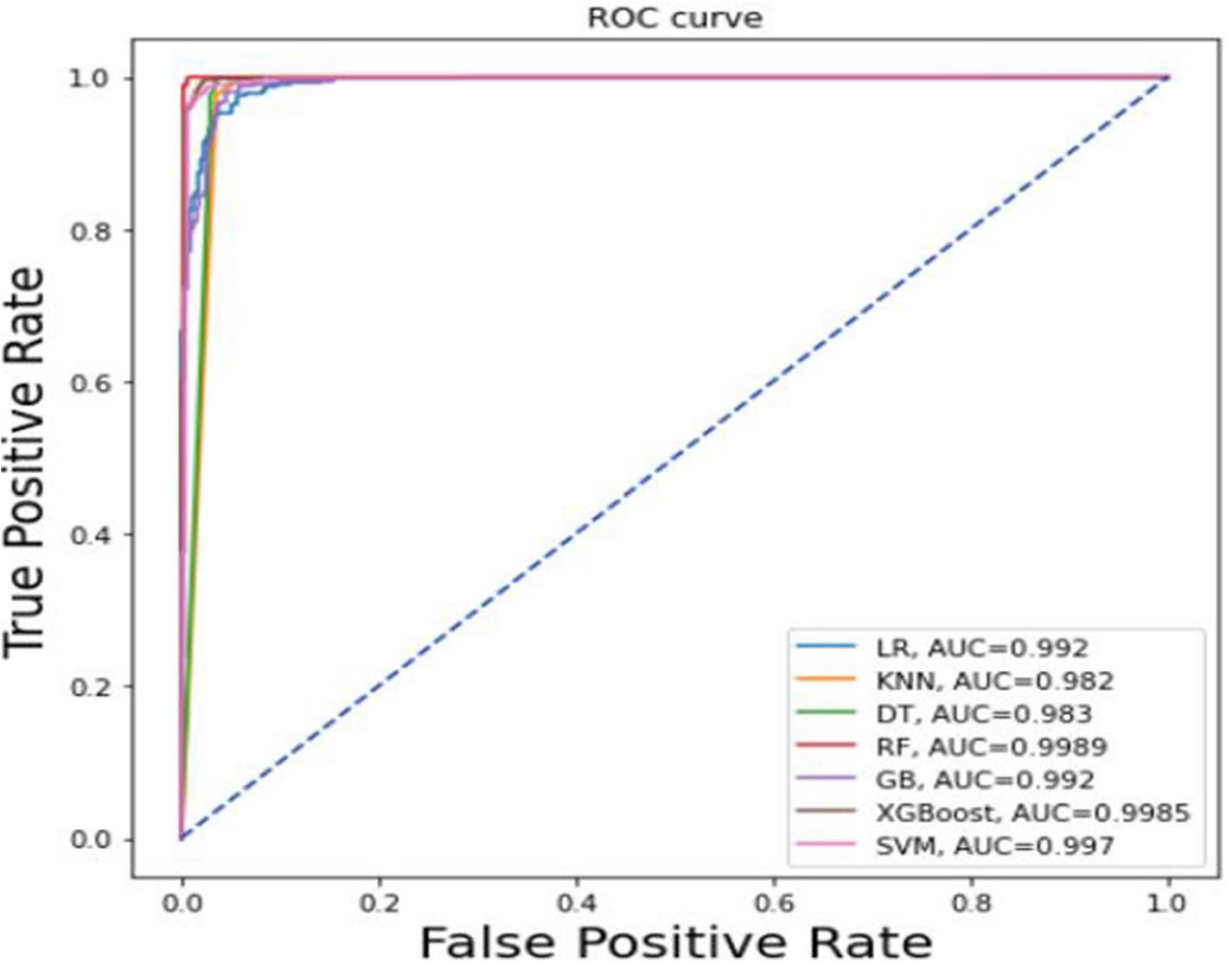
ROC curve shows a balanced dataset using SMOTE

The second half of this work was mainly concentrated on building the classification algorithm with a balanced dataset by utilizing different balancing techniques, including under-resampling, SMOTE, and ADASYN. As shown in Table 3, from all the balanced sampling methods, SMOTE was selected by comparing the AUC results for this study. After applying different machine learning classification algorithms to the imbalanced data using SMOTE, high accuracy, precision, sensitivity, specificity, f1-score, and AUC were obtained. In comparison to other algorithms, the random forest classifier produces the most accurate and meaningful results by comparing the performance of the model using performance metrics. For each method, all hyperparameters were left at their default values while the experiments were being conducted. The accuracy, precision, sensitivity, specificity, and f1-score were used in this study to assess how well the models performed. The ROC curve in Fig. 6 shows that the random forest classifier (AUC = 0.9989) outperformed all other classifiers, followed by the XGBoost classifier (AUC = 0.9985), support vector machine (AUC = 0.997), logistic regression (AUC = 0.992), gradient boosting classifier (AUC = 0.992), the k-nearest neighbor classifier (AUC = 0.982) and decision tree classifier (AUC = 0.983). The relationship between false positive and true positive rates is shown in the ROC curve, which states that a predictive model was more accurate for the prediction of virological failure (Fig. 6).

**Fig. 7:**
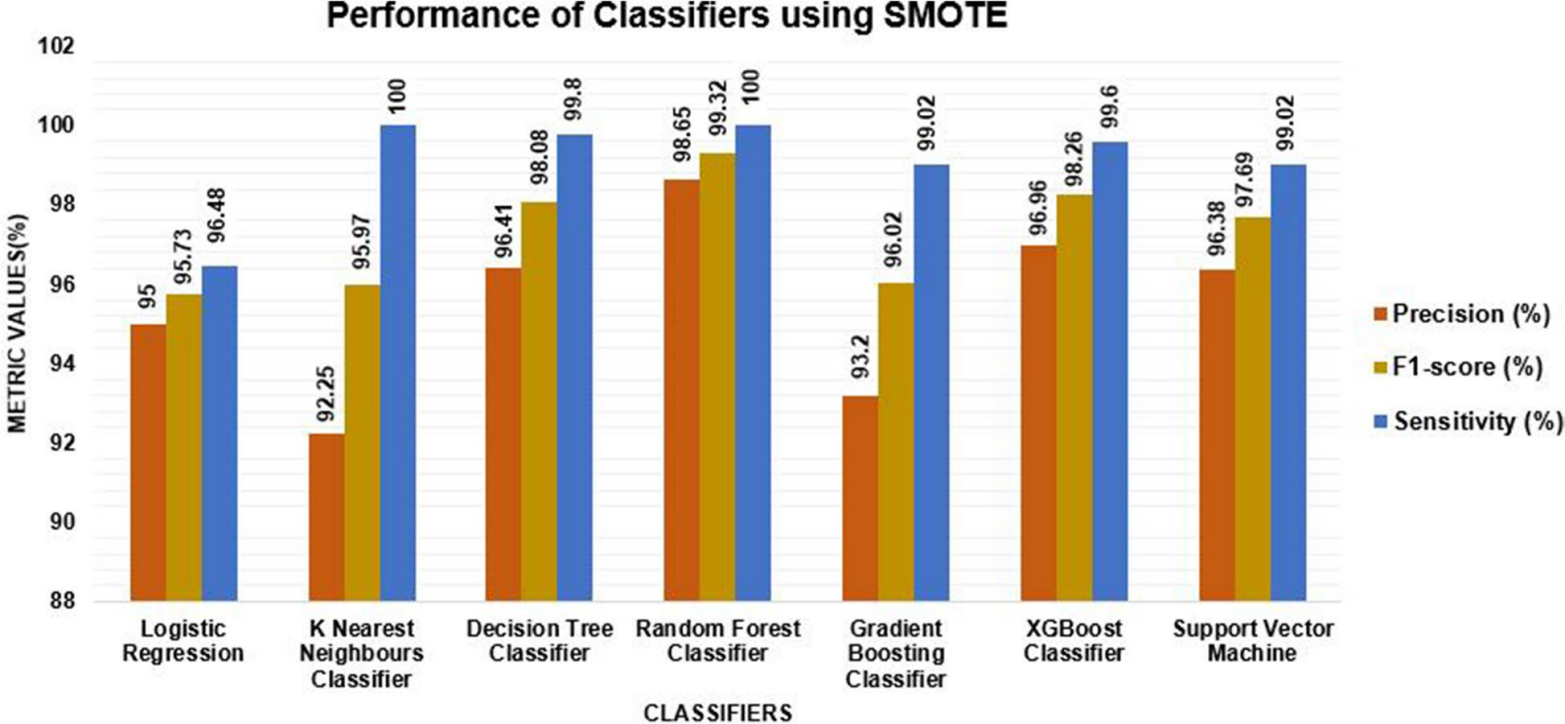
Performance of Classifier using SMOTE

The performance evaluation results with the balanced dataset in Fig. 7 show that all the classifiers of the models achieved different results. The random forest classifier achieved a sensitivity of 100%, a precision of 98.65%, and an f1-score of 99.32%. The XGBoost classifier was performed with a sensitivity of 99.60%, a precision of 96.96%, and an f1-score of 98.26%. The decision tree classifier achieved a sensitivity, precision, and f1-score of 99.80%, 96.41%, and 98.08%, respectively. The K-Nearest Neighbors Classifier achieved the highest sensitivity performance but lower precision and f1-score as compared to the random forest classifier. Support vector machine (sensitivity = 99.02%, precision = 96.38%) achieved the performance metrics; logistic regression with a sensitivity of 96.48% and precision of 95.00%, gradient boosting classifier (sensitivity = 99.02%, precision = 93.02%, f1-score = 96.02%).

Generally, from the performance metrics shown in Fig. 7, random forest achieved the highest sensitivity, precision, and f1-score. Of all the classifiers, the k-nearest neighbor classifier, the decision tree classifier, and the random forest classifier had the highest sensitivity value, which means the classifier was correctly classified as true positive. Overall, it is believed that the classifiers have an acceptable performance (Fig. 7).

### Random Forest Model performance

This experiment was developed to evaluate how well different classifiers predicted virological status. The objective of this analysis is to evaluate the accuracy of the predictions provided by the selected classifier. From the balanced dataset, the random forest classifier’s performance was quite strong compared to other selected classifiers. The hyper-parameter tuning and feature selection were carried out after the best model had been chosen. The important predictor of viral failure was established to compare the model’s performance.

### Random forest with hyperparameter tuning

**Fig. 8:**
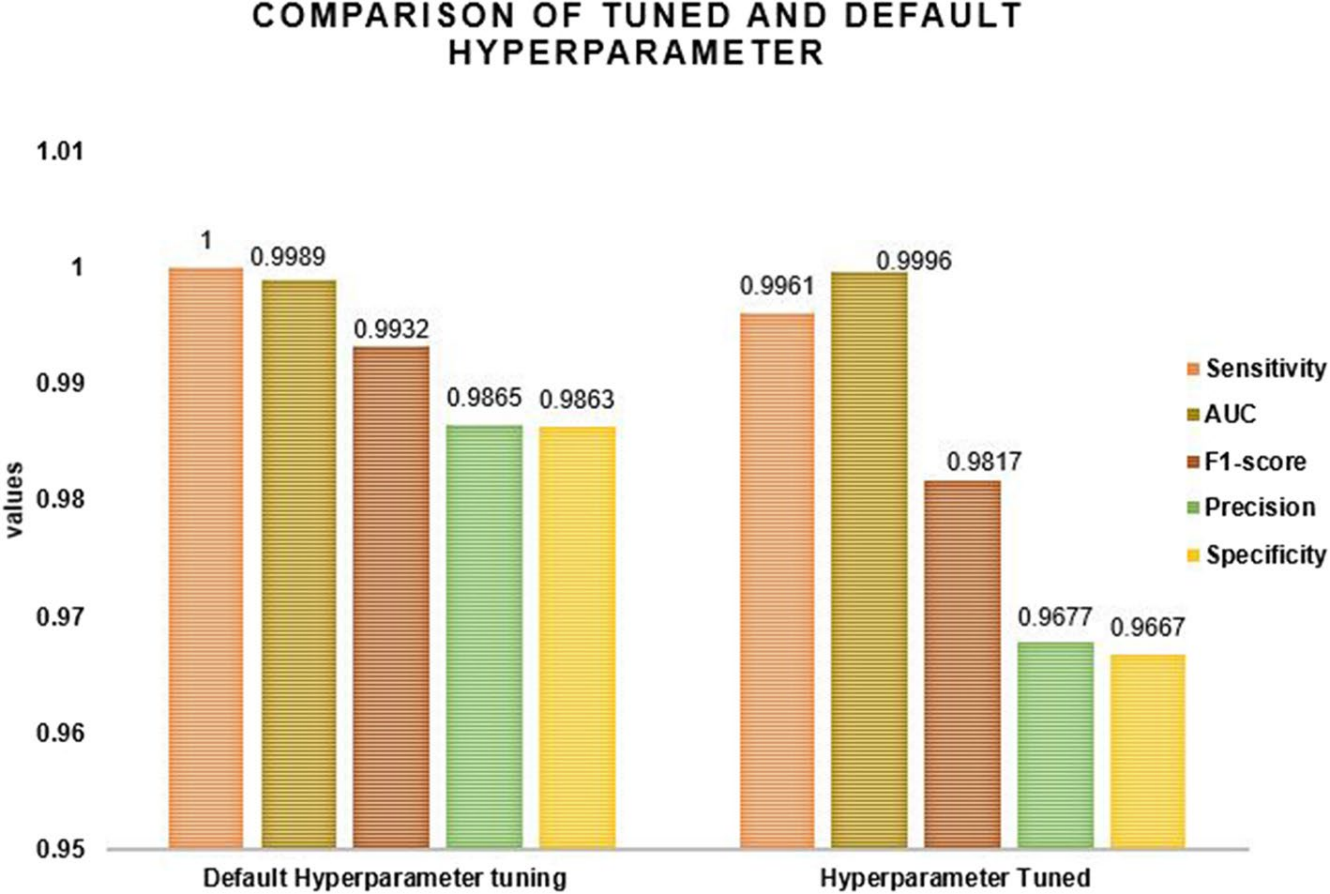
Comparison of tuned and default hyperparameter using random forest classifier

After selecting the best classifier, this study applied the hyperparameter tuning or optimation to compare it with the default hyperparameter tuning. Figure 8 shows that default hyperparameter tuning was higher performed than hyperparameter tuning using the best classifier of random forest. According to the results, the random forest classifier with tuned hyperparameters was less performed than the random forest with the default hyperparameter. Therefore, this study used a random forest with a default hyperparameter. A random forest with hyperparameter tuning of precision of 96.77%, f1-score 98.17%, the sensitivity of 99.61%, and specificity of 96.67%, as well as a random forest with default hyperparameter tuning of sensitivity, specificity, precision, and f1-score of 100%, 98.63%, 98.65%, and 99.32%, respectively. However, the random forest classifier with hyperparameter tuning surpassed all other classifiers in terms of AUC. The random forest classifier with default hyperparameter tuning had the highest sensitivity values, which means that the classifier properly identified viral unsuppressed or failed. Then this study applied the default hyperparameter tuning (Fig. 8).

### Random Forest with selected features

**Fig. 9:**
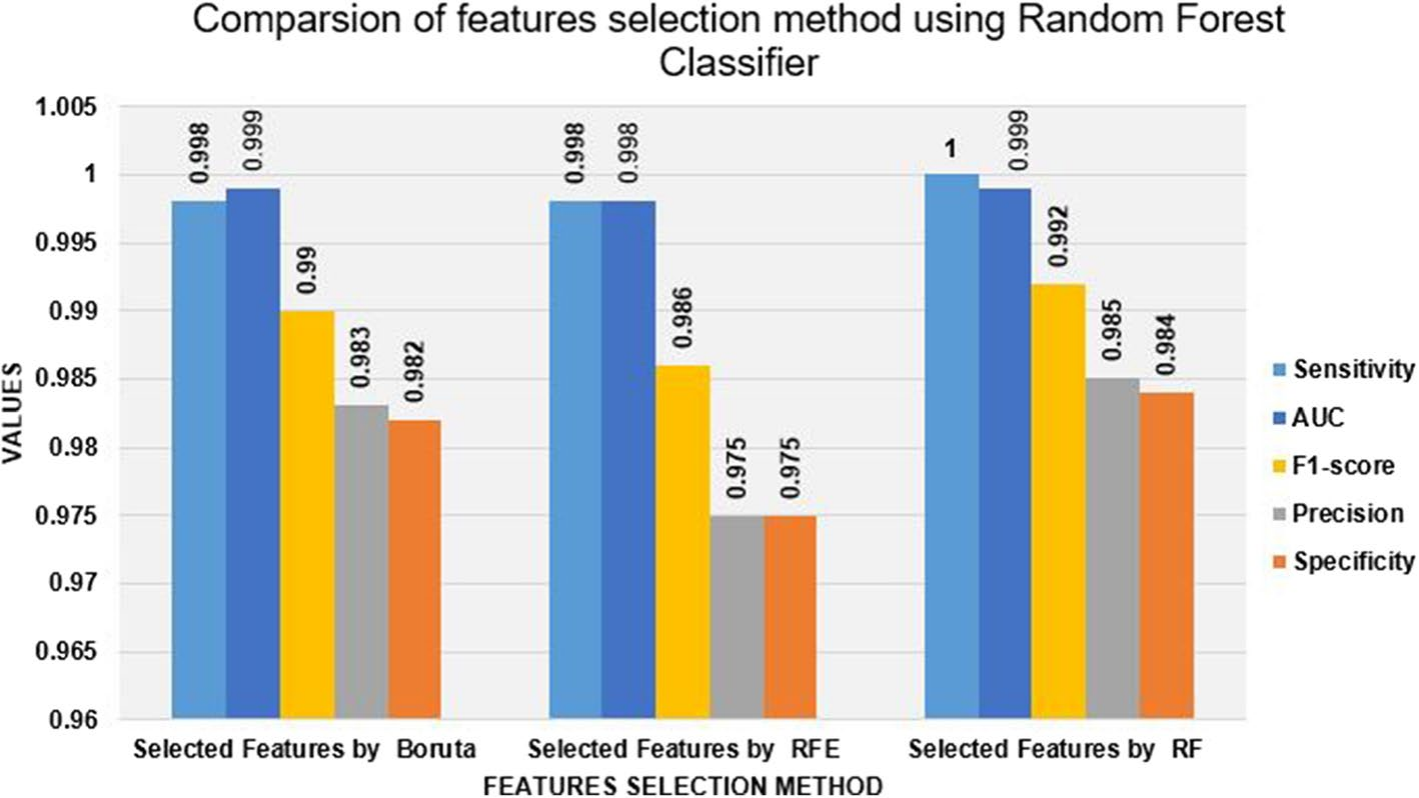
Comparison features selection method using random forest classifier

This experiment was performed to examine the random forest classifier’s ability to predict virological failure and the impact of feature selection. Different Features selection methods were applied to compare the performance of the random forest classifier. Figure 9 shows that from the features selection method, random forest features importance was performed better than both Boruta feature selection and recursive feature elimination with a sensitivity of 100%, specificity of 98.4%, a precision of 98.5%, f1-score of 99.2%, and an AUC of 0.999.

**Fig. 10:**
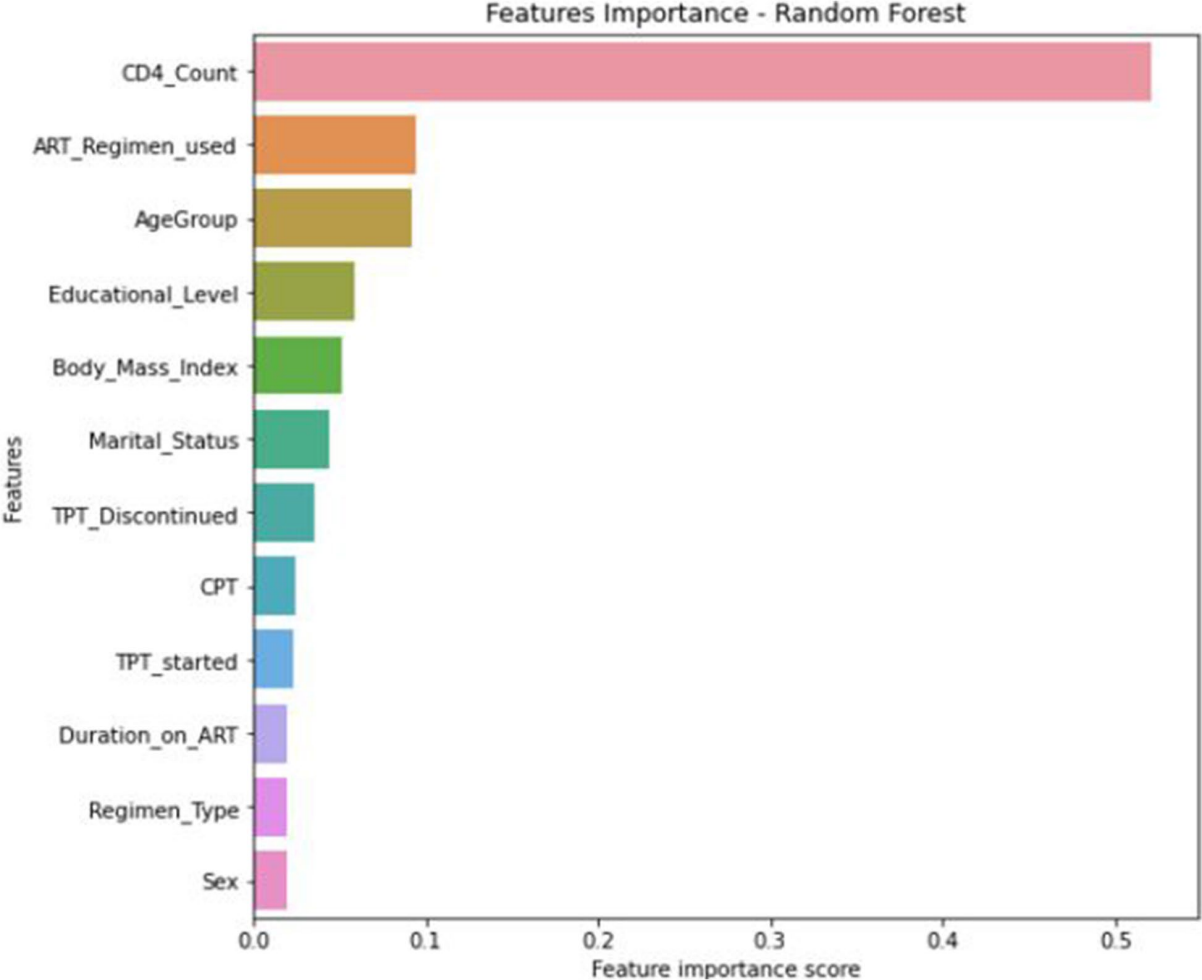
Relevant features selected by a random forest feature importance

Using these findings as a reference, for this study, random forest feature importance was chosen as the most appropriate method to select the features that most accurately predict virological failure. From the selection method, both Boruta feature selection and recursive feature elimination were less outperformed than random forest feature importance. As a result, this study used a random forest classifier as the feature importance selection method. Of all the features, CD4 count, ART regimen, age group, educational level, marital status, TPT discontinued, body mass index, regimen types, TPT started, duration of ART, sex, and CPT were highly significant for virological failure (Fig. 10).

### Association rule result

**Fig. 11:**
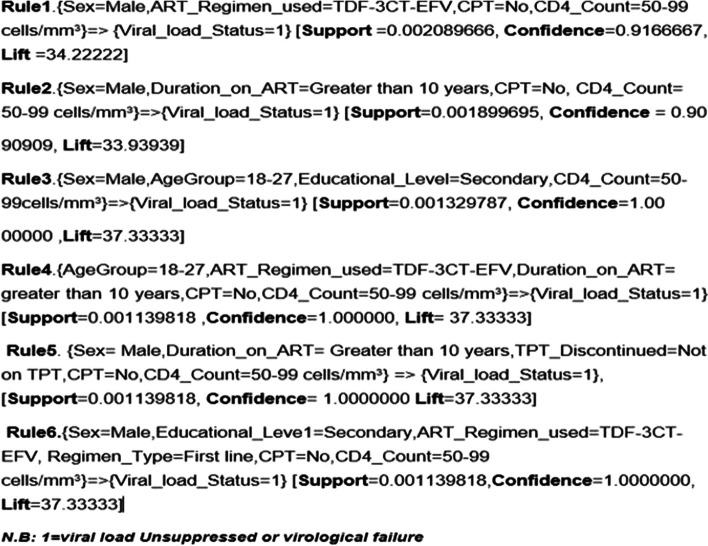
Six important association rules

After selecting the relevant features by random forest feature importance, this study applied the association mining rules with an apriori algorithm for interpretation and a comparison of the best-chosen features. Then, from the association mining rules, nine association rules were found with more than 90% confidence value and the highest lift or interestingness. Of all the rules, the six most important rules were selected for predicting virological failure **(**Fig. 11).

Rule 1 indicated that the studied client’s sex was male, the ART regin used TDF-3TC-EFV, has not taken Cotrimoxazole preventive therapy and the CD4 count of the patient had between 50 and 99 cells/mm^3^ then the possibility of developing virological failure is 92% of confidence.

Rule 2 means that being male, the client lived with ART for more than 10 years, did not take Cotrimoxazole preventive therapy and the CD4 count of the client was between 50 and 99 cells/mm^3^ then the client will a 91% chance of developing the virological failure.

Rule 3 means that if, a client whose sex was male and age was between 18 and 27, had a secondary educational level, has taken a first-line regimen, and had a CD4 count client had between 50 and 99 cells/mm^3^ then the probability of developing virological failure is 100% of confidence.

Rule 4 showed that if a client’s age was between 28 and 37, the ART regimen used TDF-3TC-EFV, the patient lived with ART for more than 10 years, not taking Cotrimoxazole preventive therapy and the CD4 count of the client was between 50 and 99 cells/mm^3^ then the possibility of developing virological failure is 100% of confidence.

Rule 5 means that if, a client whose sex was male and the client lived with ART for more than 10 years, has not taken Tb preventive therapy, and the CD4 count of the client was between 50 and 99 cells/mm^3^ then the client will be 100% confidence of developing the virological failure.

Rule 6 indicated that the studied clients’ sex was male, had a secondary educational level, have taken a first-line regimen of TDF-3TC-EFV, has not taken Cotrimoxazole preventive therapy and the CD4 count of the client was between 50 and 99 cells/mm^3^ then the possibility of developing virological failure is 100% of confidence.

Based on Association Rules results showed that males, aged between 18 and 27 and 28–37, secondary educational level, TDF-3TC-EFV, lived with ART for more than 10 years, not used TPT and CPT and CD4 count between 50 and 99 cells/mm^3^ were the most predictors of virological failure for this study. According to the above association rules, the independent and dependent features were strongly associated with one another because the lift values were more than one value.

## Discussion

Ethiopia is stepping up efforts to find patients who need more services and interventions to meet the 95-95-95 targets at each stage of treatment [7]. The goal of this study has been to predict and identify the predictors of virological failure and build the best performance of a supervised machine learning classifier. Seven supervised machine learning classification algorithms such as Logistic Regression, K-Nearest Neighbors Classifier, Decision Tree Classifier, Random Forest Classifier, Gradient Boosting Classifier, XGBoost Classifier, and Support Vector Machine, were applied to predict virological failure in patients receiving antiretroviral therapy (ART), using electronic demographic, clinical, and treatment data.

To improve model prediction accuracy and generalizability, the above models were chosen to develop and verify the best predictive model using the important predictors. The classifiers have been trained on a set of training samples by splitting stratified 10-fold cross-validation with default hyperparameter tuning. Various experiments were done to find the best accuracy using both unbalanced and balanced datasets. The result showed that unbalanced data achieved low-performance metrics. Thus, this study compared imbalanced data handling techniques. A SMOTE balancing sampling technique was selected based on the performance of accuracy and AUC. The random forest classifier outperformed all other chosen classifiers with a performance evaluation of accuracy (99.31%), sensitivity (100%), precision (98.65%), f-1-score (99.32%), and AUC (0.999). As the result, this classifier was selected in our study for the prediction of virological failure. Despite the availability of limited research on virological failure with a random forest classifier, we have attempted to compare some related research on predicting HIV/AIDS status and virological response. The results showed that the random forest classifier had a better prediction performance on virological failure. This result is supported by the previous research study conducted in Switzerland in 2020 [57]. However, there was a variation in the performance evaluation metrics between our findings and the findings of Switzerland. A possible explanation might be due to the size of the dataset. A study conducted in Switzerland used a small dataset, while our study used relatively large datasets. The small dataset might affect the performance of the predictive model [76]. Another possible difference might be due to the number of features used. This study used twenty predictors, while the study conducted in Switzerland used seven predictors. The predictors could have an impact on how well the predictive model performs [55]. The study conducted in North Carolina, USA, suggested that using a random forest classifier can predict a patient’s probability of delayed linkage to care [77]. A study using a data mining application revealed that random forest outperformed predicting CD4 change [78].

This study determined the significant influencing factors for virological failure using the relevance values of independent features for the random forest classifier. Using the random forest feature selection method, the most important predictors that contribute to better performance in virological failure prediction were identified. Among all independent features, sex, age, CPT use, ART regimen used, duration of ART, TPT discontinued, educational status, and CD4 count were the significant predictors of virological failure.

Sex was a highly significant factor in predicting virological failure. Accordingly, male HIV patients were more likely to develop virological failure as compared to females. This study is in line with the research studies conducted in Kumasi, Ghana [26], Morocco [79], Dar Es Salaam, Tanzania [36], and the Tigray region, Ethiopia [80] that stated that virological failure was more common in male HIV patients than female HIV patients. The possible reasons might be excessive alcohol consumption, low clinic attendance for ART, and low health-seeking behavior in male patients [31, 36, 80].

The machine learning classifier identified that age was a highly important feature for predicting virological failure. Accordingly, younger HIV patients are more likely to develop virological failure than their counterparts. This finding is similar to the studies conducted in Mozambique [81], in localities of Ethiopia, Gondar [82], Kombolcha town [33], and Dessie [34], which reported that virological failure occurs more frequently in younger HIV patients than in older HIV patients. The possible reason could be the emotional instability of young HIV patients, which contributes to depression and a worry about telling their families, and friends, which has a negative impact on treatment outcomes [33, 34]. This study showed that educational level was significantly associated with virological failure. The patients who were educated to a secondary level were more likely to develop virological failure. These findings were supported by research conducted in Woldia, Ethiopia [83] and North West Ethiopia [84] and the study conducted on virologic outcomes in early antiretroviral treatment [85] reported that HIV patients who were educated to a secondary level had a significant association with virological failure. The possible reasons might be the level of counseling provided to educated patients who are assumed to know better, as well as the detrimental effects of social stigma and discrimination on educated individuals [83].

Duration on ART was an important feature for predicting virological failure. Patients who had a longer duration of stay on ART had a higher risk of developing virological failure. similarly, previous studies conducted in Waghimra, Northern Ethiopia, and Mettu, South West Ethiopia [22, 86] revealed that patients who had been on ART for a longer time were more susceptible to developing virological failure. The possible explanation might be due to the prolonged use of ART, which can increase the risk of developing poor adherence to treatment and unfavorable medication reactions [26].

Based on the finding of this study, the CD4 count of the respondent was found another relevant feature for predicting virological failure. As a result, patients who had low CD4 counts were more likely to develop virological failure. This finding is supported by research conducted in the Waghimra zone, Northern Ethiopia [86], Kombolcha town [33], Dessie, Northeast Ethiopia [87], and central Oromia [88], which reported that patients with low CD4 counts were more likely to experience virological failure. The effect of low CD4 counts makes patients more susceptible to opportunistic infections, which could increase the chance of virological failure [49].

Other relevant features of virological failure were co-trimoxazole preventive therapy and Tb preventive therapy. This study showed that patients who had not taken the Cotrimoxazole preventive therapy had a high chance of developing virological failure. The finding of this study is in line with the research conducted in Kenya [89] and the Amhara region of Ethiopia [90], which indicated that patients who have not received the Cotrimoxazole preventative medication are most susceptible to developing virological failure. HIV prevention drugs, such as Cotrimoxazole preventive therapy significantly reduce morbidity and mortality rates among patients with HIV [21]. However, the study conducted in China reported that using Cotrimoxazole preventive therapy was more likely to increase virological failure [91]. The possible explanation might be due to the cut-off point of the viral load. A study conducted in China showed a viral load cut-off point of greater than 400 copies/ml two or more consecutive times after 6 months of ART, while our study used a viral load cut-off point of greater than 1000 copies/ml two or more consecutive times after three to six months of ART. This research reveals that patients who have not taken TB preventive therapy are more likely to develop virological failure. The possible explanation might be that TPT plays a role in decreasing mycobacterium load and reducing the progression of latent bacilli to active TB. The increased mycobacterial load was linked to progressive impairment of mycobacterium-specific T cell response as well as an increase in the occurrence of active TB [92]. TB is an opportunistic infection for HIV patients, those patients are more likely to develop virological failure [93].

The ART regimen was highly important for predicting virological failure. This research study showed that patients who were taking the first-line ART regimen of TDF-3TC-EFV were more likely to develop virological failure. Findings from a research study which was conducted in Nepal [94] and Namibia [95] supported that patients who received the first-line ART regimen of TDF-3TC-EFV were more prone to virological failure. This idea is also supported by the WHO, which recommends starting ART treatment with DTG rather than EFV due to difficulties with the high prevalence of depression, dizziness, and treatment-related adverse effects [21].

In conclusion, our study demonstrates the feasibility of using machine learning methods to identify HIV patients at high risk of virologic failure and to determine predictive factors associated with virologic failure. Machine learning algorithms seem to work effectively for risk prediction and classification of ART treatment failure, but further refinements are needed. Nevertheless, our study model may contribute to the important public health issue of reaching and treating HIV-infected patients.

Machine learning can be a great help to clinicians who care for HIV patients. The proposed algorithm can predict viral failure in HIV patients with optimal ROC, accuracy, precision, sensitivity, and specificity. This prediction helps to optimize the use of hospital resources to treat high-risk patients, provide better quality care, and reduce medical errors caused by fatigue and long work hours in ART clinics. Designing effective predictive models may improve the quality of care and improve patient survival. Therefore, our study of predictive models of viral failure can make a significant contribution to identifying HIV patients at high risk of viral failure and adopting the most effective supportive and therapeutic regimens. This may reduce ambiguity by providing quantitative, objective, and evidence-based models for risk stratification, prediction, and ultimately care planning. In addition, the findings of this study may provide clinicians with better strategies to reduce complications and improve the chances of survival of HIV patients.

### Strengths and Limitations of the study

One of the strengths of this work is that it incorporates seven supervised machine-learning classification algorithms. Despite its strength, this study is not without limitations. This study was performed in a single-site ART treatment registry database, which may limit the generalizability of the developed model. However, the dataset used in this study is a database collected at Gondar University Hospital, which provides specialized ART treatment services for HIV patients. Data were collected retrospectively, some data were incomplete or missing, and behavioral data such as smoking, alcohol consumption, exercise, and dietary habits were not analyzed in the study. For handling missing data, this study used simple imputation techniques. Moreover, this study used data that was unbalanced, meaning that the majority class and the minority class of dependent features were unequally distributed.

## Conclusion

This study suggests that the random forest classifier outperformed based on the performance evaluation metrics. Male, younger age, longer time on ART, not taking CPT, not taking TPT, secondary educational level, TDF-3TC-EFV, and low CD4 counts were significant features for predicting virological failure.

## Data Availability

The datasets used during the current study are available from the corresponding author upon reasonable request.

## Abbreviations

3TC: Lamivudine
AIDS: Acquired Immunodeficiency Syndrome
ART: Antiretroviral Therapy
AUC: Area Under Curve
AZT/ZDV: Zidovudine
BMI: Body Mass Index
CD4: Cluster Differentiation 4
CPT: Cotrimoxazole Prophylaxis Therapy
CSV: Comma Separated Values
DTG: Dolutegravir
DRV/r: Ritonavir boosted Darunavir
EFV: Efavirenz
HIV: Human Immunodeficiency Virus
MRN: Medical Record Number
RNA: Ribonucleic Acid
SNNP: South Nation Nationality People
UNAIDS: United Nations Programme on Acquired Immune Deficiency Syndrome
WHO: World Health Organization
XGBoost: eXtreme Gradient Boosting
