# Correction: Machine learning to predict virological failure among HIV patients on antiretroviral therapy in the University of Gondar Comprehensive and Specialized Hospital, in Amhara Region, Ethiopia, 2022

**DOI:** 10.1186/s12911-025-02908-w

**Published:** 2025-02-25

**Authors:** Daniel Niguse Mamo, Tesfahun Melese Yilma, Makda Fekadie Tewelgne, Yakub Sebastian, Tilahun Bizuayehu, Mequannent Sharew Melaku, Agmasie Damtew Walle

**Affiliations:** 1 Department of Health Informatics, College of Medicine and Health Sciences, School of Public Health, Arbaminch University, Arbaminch, Ethiopia; 2 Department of Health Informatics, Institute of Public Health, University of Gondar, Gondar, Ethiopia; 3 College of Engineering, IT, and Environment, Charles Darwin University, Casuarina, Australia; 4 Department of Internal Medicine, School of Medicine, University of Gondar, Gondar, Ethiopia; 5 Department of Health Informatics, college of health science, Mettu University, Mettu, Ethiopia

**Correction: Mamo et al.**

**BMC Medical Informatics and Decision Making (2023) 23:75**

10.1186/s12911-023-02167-7.

Following the publication of the original article, the authors reported an error in the spelling of the third author’s name.

**Incorrect**.

Makida Fekadie.

**Correct**.

Makda Fekadie Tewelgne.

This correction shows the correct name of the author.

The original article has been corrected.

